# Mitochondrial efficiency and exercise economy following heat stress: a potential role of uncoupling protein 3

**DOI:** 10.14814/phy2.13054

**Published:** 2017-02-08

**Authors:** Roy M. Salgado, Ailish C. Sheard, Roger A. Vaughan, Daryl L. Parker, Suzanne M. Schneider, Robert W. Kenefick, James J. McCormick, Nicholas P. Gannon, Trisha A. Van Dusseldorp, Len R. Kravitz, Christine M. Mermier

**Affiliations:** ^1^ Department of Health, Exercise and Sports SciencesUniversity of New MexicoAlbuquerqueNew Mexico; ^2^ School of Kinesiology and Nutritional ScienceCalifornia State University Los AngelesLos AngelesCalifornia; ^3^ Department of Exercise ScienceHigh Point UniversityHigh PointNorth Carolina; ^4^ Deparment of Kinesiology and Health ScienceCalifornia State University SacramentoSacramentoCalifornia; ^5^ Thermal and Mountain Medicine DivisionUnited States Army Research Institute of Environmental MedicineNatickMassachusetts; ^6^ School of MedicineMedical College of WisconsinMilwaukeeWisconsin; ^7^ Department of Exercise and Sport ManagementKennesaw State UniversityKennesawGeorgia

**Keywords:** Altitude, cross‐tolerance, exercise capacity, heat acclimation, mitochondria

## Abstract

Heat stress has been reported to reduce uncoupling proteins (UCP) expression, which in turn should improve mitochondrial efficiency. Such an improvement in efficiency may translate to the systemic level as greater exercise economy. However, neither the heat‐induced improvement in mitochondrial efficiency (due to decrease in UCP), nor its potential to improve economy has been studied. Determine: (i) if heat stress in vitro lowers UCP3 thereby improving mitochondrial efficiency in C2C12 myocytes; (ii) whether heat acclimation (HA) in vivo improves exercise economy in trained individuals; and (iii) the potential improved economy during exercise at altitude. In vitro, myocytes were heat stressed for 24 h (40°C), followed by measurements of UCP3, mitochondrial uncoupling, and efficiency. In vivo, eight trained males completed: (i) pre‐HA testing; (ii) 10 days of HA (40°C, 20% RH); and (iii) post‐HA testing. Pre‐ and posttesting consisted of maximal exercise test and submaximal exercise at two intensities to assess exercise economy at 1600 m (Albuquerque, NM) and 4350 m. Heat‐stressed myocytes displayed significantly reduced UCP3 mRNA expression and, mitochondrial uncoupling (77.1 ± 1.2%, *P* < 0.0001) and improved mitochondrial efficiency (62.9 ± 4.1%, *P* < 0.0001) compared to control. In humans, at both 1600 m and 4350 m, following HA, submaximal exercise economy did not change at low and moderate exercise intensities. Our findings indicate that while heat‐induced reduction in UCP3 improves mitochondrial efficiency in vitro, this is not translated to in vivo improvement of exercise economy at 1600 m or 4350 m.

## Introduction

Existing evidence suggests that heat acclimation (HA) may improve economy of exercise, defined as a reduction in oxygen uptake for a given work‐rate in both hot and temperate environments (Shvartz et al. 1977; Jooste and Strydom 1979; Sawka et al. 1983). This improved economy is thought to be the result of an improvement in the efficiency of oxidative phosphorylation at the cellular level (Sawka et al. 1983). The proposed mechanism for HA‐induced improvements in oxidative phosphorylation may be a reduction in expression of mitochondrial uncoupling proteins (UCPs), which allow proton leakage across the intermitochondrial membrane, thus reducing phosphorylation efficiency, and increasing the energy “wasted” as heat. In skeletal muscle, the predominant UCP isoform is UCP3.

Previous reports of in vitro and in vivo studies suggest that UCP3 knockout mice have improved mitochondrial oxidative efficiency (Vidal‐Puig et al. 2000; Cline et al. 2001). In heat exposed chickens, Mujahid et al. (2007, 2006) reported that heat stress (34°C, 18 h) lowered expression of avian UCP in the mitochondria of skeletal muscle. However, they did not measure mitochondrial oxidative efficiency. In humans, 1 h of exercise combined with 3 h of passive heat exposure (33 and 38°C) had little effect on altering UCP3 mRNA expression (Slivka et al. 2012; Dumke et al. 2013). However, it is unclear if longer exposure, as in the studies by Mujahid et al. (2007, 2006), and/or greater heat stress, would induce a reduction in UCP3 and mitochondrial oxidative efficiency. It is also unknown if improved mitochondrial efficiency would translate to improved exercise economy at the systemic level.

Early studies support the idea that HA induces an improvement in exercise economy (Strydom et al. 1966; Jooste and Strydom 1979; Sawka et al. 1983, 1985). Shvartz et al. (1977) showed that after 8 days of HA there was a ~10.8–13.5% improvement in exercise economy during step‐test exercise. Jooste and Strydom (1979) used a 7 day HA protocol (31°C, 4 h per day) and reported a lower exercise economy during treadmill exercise. Using treadmill exercise, Sawka et al. (1983) showed a 3–7% improvement in economy after a 10‐day HA. However, these findings are inconsistent as Young et al. (1985) later found that 9 days of HA (49°C, 20% RH) only lowered exercise economy by 1%.

The purpose of the study was threefold: (i) to investigate the effects of 24‐h heat stress (40°C) on mitochondrial efficiency and UCP3 expression in C2C12 murine myocytes; (ii) to determine if HA improves cycle exercise economy in trained humans; and (iii) to determine if a potential improvement in exercise economy would be beneficial during cycling exercise in a hypobaric environment. While not identical in design to our humans studies, our goal in using a cellular model was to investigate potential mechanisms of adaptations that may contribute to previously reported improvements in exercise economy following HA. Additionally, the findings of this study may have important application for exercise training and as a means to prepare individuals who work (military, alpine workers) or perform recreational activities (athletes, mountaineers, and recreational hikers) at altitude.

## Methods and Materials

**Figure 1:**
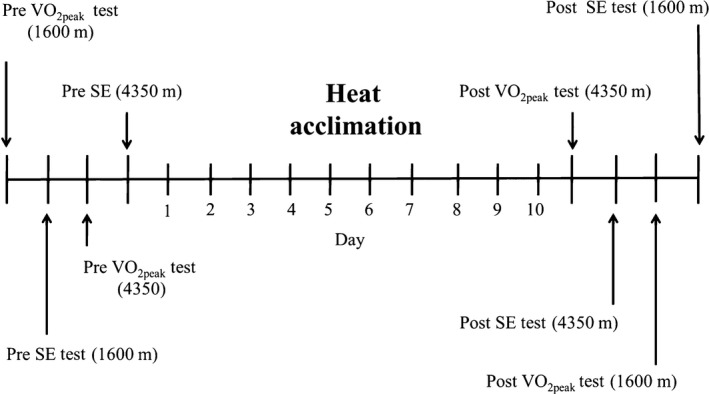
Schematic of pretesting, 10 day heat acclimation, and posttesting. Each pre and posttest was separated by at least 24–48 h. V0_2pcak_, Peak oxygen uptake; SE, Submaximal exercise test.

To address the cellular research questions, assays of cellular metabolism were performed after 24 h of heat stress to determine oxidative metabolism, mitochondrial uncoupling and mitochondrial efficiency. Then, assays for expression of UCP3, markers of mitochondrial biogenesis and mitochondrial content were performed. In the human experiments, each volunteer completed three phases over ~5 weeks (Fig. 1). These phases consisted of: (i) pre‐HA testing; (ii) 10 day HA; and (iii) post‐HA testing. Pretesting consisted of anthropometrics (height, ht and weight, wt), assessment of peak oxygen uptake (VO_2peak_) and measurement of oxygen uptake at two different submaximal workloads to determine exercise economy. Posttesting consisted of measurement of VO_2peak_ and submaximal exercise. Pre and posttesting were performed at 1600 m and 4350 m and separated by at least 24 to 48 h. After pretesting all volunteers completed a traditional 10 day HA protocol. During HA core temperature, heart rate, and perceptual measurements (RPE, thermal sensation) were measured.

### Cellular model

C2C12 murine myocytes (ATCC, Manassas, VA) were cultured in Dulbecco's Modified Eagle's Medium (DMEM, Sigma Aldrich, St. Louis, MO) in a humidified 5% CO_2_ atmosphere at 37°C (standard conditions). Following overnight seeding, cells with either incubated for 24 h under standard conditions (control) or at 40°C (5% CO_2_) (heat stressed).

### Metabolic assay

Cells were seeded overnight in 24‐well culture plate (SeaHorse Bioscience, Billerica, MA) at a density of 5 × 10^5^ cells/well, and incubated either under standard (*n* = 22 wells for control) or heat‐stressed conditions (*n* = 22 wells heat stress). Following the 24 h incubation, culture media was removed and replaced with XF Assay Media (SeaHorse Bioscience). Per manufacturers’ protocol, SeaHorse injection ports were loaded with oligomycin and carbonyl cyanide *p*‐[trifluoromethoxy]‐phenyl‐hydrazone (FCCP). Prior to any chemical treatment, basal oxidative metabolism (oxygen consumption rate, OCR) was measured. Then to reveal endogenous proton leak (mitochondrial uncoupling) cells were treated with oligomycin at a final concentration 1.0 *μ*mol/L. Finally, FCCP, an uncoupler of electron transport at a concentration of 1.25 *μ*mol/L was added to determine peak oxygen consumption (an indirect indicator of peak oxidative metabolism) (Giulivi et al. 2008; Wikstrom et al. 2012). SeaHorse XF24 Extracellular Analyzer was run using 8 min cyclic protocol commands (mix for 3 min, let stand 2 min, and measure for 3 min) in triplicate as previously performed (Vaughan et al. 2013).

### Quantitative real‐time polymerase chain reaction (qRT‐PCR)

Total RNA was extracted from control and heat stress cells, using RNeasy Kit from Qiagen (Valencia, CA) and cDNA was synthesized (Retroscript^™^ RT kit, Ambion (Austin, TX). PCR primers were designed (Primer Express software, Invitrogen, Carlsbad, CA) and synthesized by Integrated DNA Technologies (Coralville, IA). Amplification of peroxisome proliferator‐activated receptor gamma coactivator 1‐alpha (PGC‐1*α*), nuclear respiratory factor 1 (NRF1), mitochondrial transcription factor A (TFAM), and UCP3 were normalized to the housekeeping gene, TATA‐binding protein (TBP). Table 1 summarizes the forward and reverse primers of each gene; qRT‐PCR reactions were performed in triplicate for each condition (*n* = 3 wells) (Light Cycler 480 real‐time PCR; Roche Applied Science, Indianapolis, IN), using SYBR Green‐based PCR with final primer concentrations at 10 *μ*mol/L in a total volume of 30 *μ*L. The following cycling parameters were used: 95°C for 10 min, followed by 45 cycles of 95°C for 15 sec, and 60°C for 1 min. Relative expression levels were determined by the ΔΔCp method and compared to the lowest expressing group as previously described (Pfaffl 2001).

**Table 1:** Summary of qRT‐PCR primers

Primer Name	Forward sequence	Reverse sequence
NRF1	5′‐ACCCTCAGTCTCACGACTAT‐3′	5′‐GAACACTCCTCAGACCCTTAAC‐3
PGC‐1*α*	5′‐GACAATCCCGAAGACACTACAG‐3′	5′‐AGAGAGGAGAGAGAGAGAGAGA‐3′
TBP	5′‐GGGATTCAGGAAGACCACATA‐3′	5′‐CCTCACCAACTGTACCATCAG‐3′
TFAM	5′‐GAAGGGAATGGGAAAGGTAGAG‐3′	5′‐ACAGGACATGGAAAGCAGATTA‐3′
UCP3	5′‐CAGATCCTGCTGCTACCTAAT‐3′	5′‐GCATCCATAGTCCCTCTGTAT‐3′

Primers were acquired from Integrated DNA Technologies (Coralville, IA). Abbreviations: Nuclear respiratory factor 1 (NRF1), peroxisome proliferator‐activated receptor *γ* coactivator 1 alpha (PGC‐1*α*), mitochondrial transcription factor A (TFAM), TATA‐binding protein (TBP), and mitochondrial uncoupling protein 3 (UCP3).

### Immunoblotting and protein expression

Immunoblotting was done in triplicate for each condition (*n* = 3 control and *n* = 3 heat stress). Whole cell lysates were prepared by harvesting the cells on ice in high salt lysis buffer (25 mmol/L Tris base, 8 mmol/L MgCl2, 1 mmol/L DTT, 15% glycerol, 0.1% Triton) supplemented with protease inhibitor mix (Sigma Aldrich, St. Louis, MO), followed by incubation on ice for 60 min. Insoluble material was removed by centrifugation at 250 × *g* for 3 min and protein concentrations were determined by Bradford assay (Protein Assay Dye Reagent Concentrate, Bio‐Rad Laboratories, Hercules, CA). Total protein (40 *μ*g per sample) was size‐separated by 10% sodium dodecyl sulfate polyacrylamide gel electrophoresis (SDS‐PAGE) and electro‐transferred to nitrocellulose membranes. After blocking in TBST‐5% milk powder for 1 h, membranes were probed at 4°C for 24 h with an anti‐PGC‐1*α* primary polyclonal antibody from Santa Cruz Biotechnologies (Santa Cruz, CA). Bound antibodies were detected by horseradish peroxidase‐conjugated secondary antibodies from Sigma and by chemiluminescence using the ECL Plus Western Blotting Detection kit from GE Healthcare Life Sciences (Little Chalfont, Buckinghamshire, UK). Signal intensities were obtained by densitometry using ImageJ software (available from the NIH at http://rsbweb.nih.gov/ij/) by quantifying lane intensities followed by normalizing PGC‐1*α* intensity with corresponding *β*‐actin.

### Flow cytometry

Cells were seeded in 6‐well (*n* = 6 control and *n* = 6 heat stress) plates at a density of 1.0 × 10^6^ cells/well and incubated as described above. Following incubation, the media was removed and the cells were resuspended in prewarmed media with 200 nmol/L Mitotracker Green (Life Technologies, Carlsbad, CA) and incubated for 45 min in a humidified 5% CO_2_ atmosphere at 37°C. The cells were pelleted, the media with Mitotracker was removed and the cells were suspended in prewarmed media. Group mean fluorescence was measured using Facscalibur filtering 488 nm.

### Human model

## Subjects

Eight trained male cyclists and runners (age: 28 ± 6 years, ht: 1.78 ± 0.08 m, wt: 75.7 ± 8.4 kg and VO_2peak_: 4.19 ± 0.54 L/min; mean ± SD) were recruited to participate in this study. All volunteers resided at altitudes of 1500–1600 m at least 6 months prior to the study. Testing was performed at Albuquerque, NM (1600 m, 633 mmHg) during February 2014 to June 2014 with an average outside temperature of 23.6°C. Written informed consent as approved by the University Human Research Review Committee at the University of New Mexico was obtained prior to participation in the study.

#### Maximal exercise test

The maximal exercise test on a cycle ergometer (Velotron DynaFit Pro, RacerMate, Seattle, WA) began at 70 W for 1 min and workload increased 35 W every minute until volunteers could not maintain 60 rpm. Heart rate (HR) (Polar Electro, model FS1, Woodbury, NY), saturation of oxygen (SaO_2_) (GO_2_ Pulse Oximeter, Philips Respironics, Andover, MA) and rating of perceived exertion (RPE; 6–20 scale), were recorded every minute. Breath‐by‐breath gas exchange was measured continuously (ParvoMedics True One 2400; Sandy, Utah) with VO_2peak_ recorded as the highest average value over any 15‐sec period.

Peak power output was defined as the highest workload in W from the last completed 1‐min stage plus the fraction of time spent in the uncompleted final workload multiplied by 35 W (Stepto et al. 1999). Ventilatory threshold (VT) was determined by an increase in Ve/VO_2_ with no change in Ve/VCO_2_ (Davis 1985), while the respiratory compensation point (RCP) was defined as simultaneous increase in Ve/VO_2_ and Ve/VCO_2_ (Takano 2000).

#### Submaximal exercise tests

Two 30 min submaximal exercise bouts were performed in temperate conditions (21°C) at 1600 m and at 4350 m. The initial 10 min consisted of a warm‐up at a self‐selected workload. Immediately following the warm‐up, two 10 min exercise bouts were performed at low and moderate intensities. Adjusted workloads were determined for each individual by subtracting 75 W from the power at VT (1600 m: 171 ± 44 W and 4350 m: 133 ± 32 W) (Bradley, RT 2012 unpubl obser.). Then ~70 and ~80% of the adjusted workload was calculated to determine low and moderate intensities. The average workload at 1600 m was 120 ± 30 W and 137 ± 35 W; while at 4350 m was 95 ± 23 W and 108 ± 26 W for low and moderate intensity, respectively. These intensities were selected to avoid the VO_2_ slow component (Poole et al. 1994) and ensure subjects reached steady‐state. To determine changes in economy, participants exercised at the same workload before and after HA. Cadence was maintained at 80 rpm during all trials.

#### Heat acclimation

The HA protocol has been previously reported (White et al. 2016). Briefly, volunteers completed 10 consecutive days of HA at 1600 m (40°C and ~20% relative humidity). The protocol consisted of two 50‐min cycling bouts (Monark Erogmedic, Model 828E, Varberg, Sweden) separated by 10 min of passive heat exposure. To prevent a training effect (Sady et al. 1980; Londeree 1997), exercise was performed at the adjusted workload (described above, 171 ± 44 W) from the first maximal exercise test at 1600 m. Core temperature by rectal probe (Telly Themometer, Yellow Springs, OH), thermal sensation, HR and RPE were recorded every 5 min. Water intake was provided ad libitum and total volume and urine output were recorded. Changes in nude bodyweight, water intake, and urine volume were used to calculate the sweat rate.

### Statistical analyses

#### Cell model

Student's *t*‐test was used to determine differences in metabolic measurements, gene expression, protein expression, and mitochondrial content between control and heat‐stressed cells.

#### Human model

An a priori power analysis (G*Power, Universitat Kiel, Germany, version 3.1) with repeated measures was used to determine the sample size needed to find a significant change in submaximal exercise oxygen consumption. The effect size from submaximal oxygen consumption was chosen because reductions in oxygen uptake during submaximal exercise at have been associated with improved exercise tolerance at altitude. Using an effect size of 0.98 calculated from changes in submaximal oxygen consumption after HA (Shvartz et al. 1977), an alpha level of 0.05 and power of 0.80, a total of six subjects were needed for this investigation.

Dependent *t*‐test was used to determine differences in HA responses from day 1 to day 10. A three‐way (altitude × time × intensity) repeated measures ANOVA was used to determine differences in economy of exercise, metabolic (VO_2_, VCO_2_, and RER), cardiopulmonary (VE and HR), and perceptual measurements (RPE). A two‐way (altitude × time) repeated measures ANOVA was used to determine differences in the mean slope and mean *y*‐intercept of the oxygen uptake of the two different submaximal exercise intensities, VO_2peak_ and peak power. All statistical analyses were conducted, using a commercially available software (Statistica v10, Statsoft Inc., Tulsa, OK). Significance was set at *P* ≤ 0.05. Results are reported as mean ± SD.

## Results

### Cellular model

#### Changes in cellular metabolism after heat stress

**Figure 2:**
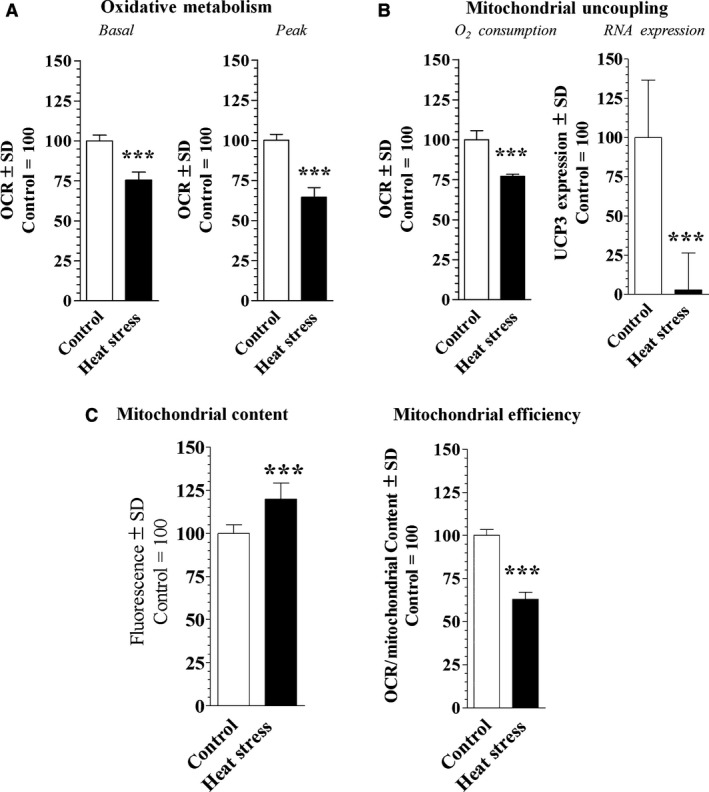
Illustrates cellular metabolism of C2C12 murine myocyte incubated under 37°C (control, *n* = 22) or 40°C (heat stressed, *n* = 22) for 24 h. (A) Basal and peak oxidative metabolism indicated by oxygen consumption rate (OCR) (B) Endogenous uncoupling revealed by oligomycin treatment of cells treated as described above and mitochondrial uncoupling protein 3 (UCP3) RNA expression. (C) Mitochondrial content indicated by Mitotracker (*n* = 6) staining measured by flow cytometry and basal oxygen consumption per relative mitochondria of cells (normalized to mitochondrial content). Significance was indicated as *, **, and **^*^ indicating *P* < 0.05, *P* < 0.01, and *P* < 0.001 statistical differences compared to control, respectively. Data were standardized to the control and reported as percentage.

Figure 2A shows that basal (75.5 ± 4.9%, *P* < 0.0001, 95% CI = 21.89 to 27.14) and peak OCR (64.4 ± 5.9%, *P* < 0.0001, 95% CI = 32.51 to 38.62) was reduced in heat‐stressed cells compared to control cells. Mitochondrial H^+^ leak (uncoupling), a source of thermogenesis, was reduced (77.1, ±1.2%, *P* < 0.0001, 95% CI = 20.3 to 25.4) in the heat‐stressed cells, and accompanied by a significantly reduced UCP3 gene expression (*P* = 0.018, 95% CI = 27.4 to 166.8) (Fig. 2B).

Heat‐stressed cells displayed significantly increased mitochondrial staining (119.9%, ± 9.2%, *P* = 0.0008, 95% CI = −29.4 to −10.5) compared with control cells (Fig. 2C) indicative of greater mitochondrial content. To investigate the effects of heat stress on mitochondrial efficiency, basal oxygen consumption was normalized to mitochondrial staining. Figure 2C shows that heat‐stressed cells also had lower oxygen consumption per relative mitochondria (62.9 ± 4.1%, *P *< 0.0001, 95% CI = 34.7 to 39.42).

#### Changes in gene expression due to heat stress

**Figure 3:**
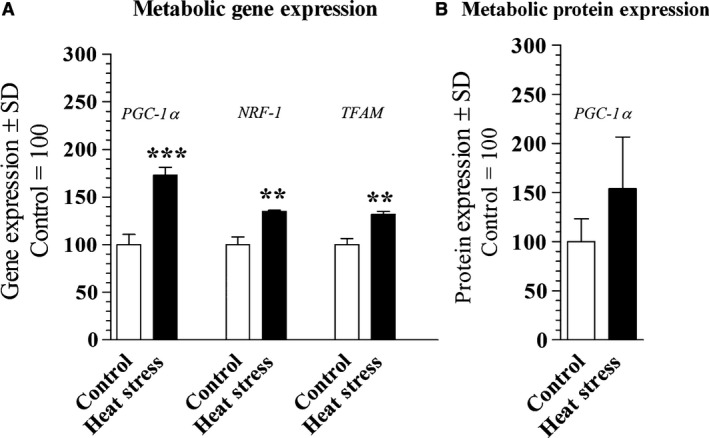
Illustrates gene and protein expression of C2C12 murine myocyte incubated under 37°C (control) or 40^°^C (heat stressed) for 24 h. (A) Metabolic gene expression (*n* = 3) of peroxisome proliferator‐activated receptor γ coactivator 1 alpha (PGC‐lα), nuclear respiratory factor 1 (NRF1), and mitochondrial transcription factor A (TFAM) were normalized to the housekeeping gene, TATA‐binding protein (TBP) and, (B) Protein expression (*n* = 3) of cells treated as described above of PGC‐lα. Significance was indicated as *, **, and *** indicating *P* < 0.05, *P* < 0.01, and *P* < 0.001 statistical differences compared to control, respectively. Data were standardized to the control and reported as percentage.

Figure 3A shows that heat‐stressed cells had increased PGC‐1*α* (*P* = 0.0007, 95% CI = −94.8 to −51.4), as well as downstream targets NRF‐1 (*P* = 0.0021, 95% CI = −48.9 to −21.4), TFAM expression (*P* = 0.0016, 95% CI = −43.7 to −20.3). However, PGC‐1*α* protein expression was not different in heat stress compared to control cells (*P* = 0.232, 95% CI = −127.2 to 41.63) (Fig. 3B).

### Human model

#### Heat acclimation

Heat acclimation responses were previously reported (White et al. 2016). Briefly, end core temperature (day 1: 39.3 ± 0.7°C vs. day 10: 38.8 ± 0.6°C, *P* = 0.042), thermal sensation (day 1: 7 ± 1 ±  vs. day 10: 6 ± 1, *P* = 0.002), end HR (day 1: 162 ± 18 bpm vs. 141 ± 15 bpm, *P* < 0.001) and RPE (day 1: 16 ± 3 vs. day 10: 13 ± 1, *P* = 0.021) were lower from day 1 to day 10, indicating HA was achieved.

#### Maximal exercise

There was an increase peak power measured from the maximal exercise test pre‐ 342 ± 50 W, 95% CI = 301 to 383) to post‐HA (353 ± 43 W, 95% CI = 317 to 388) (*P* = 0.042, *d *=* *0.22, ƞp^2^ = 0.46), although no interaction effect (altitude × time) was observed (*P* = 0.052, *P* = 0.83, ƞp^2^ = 0.007).

#### Submaximal exercise

**Figure 4:**
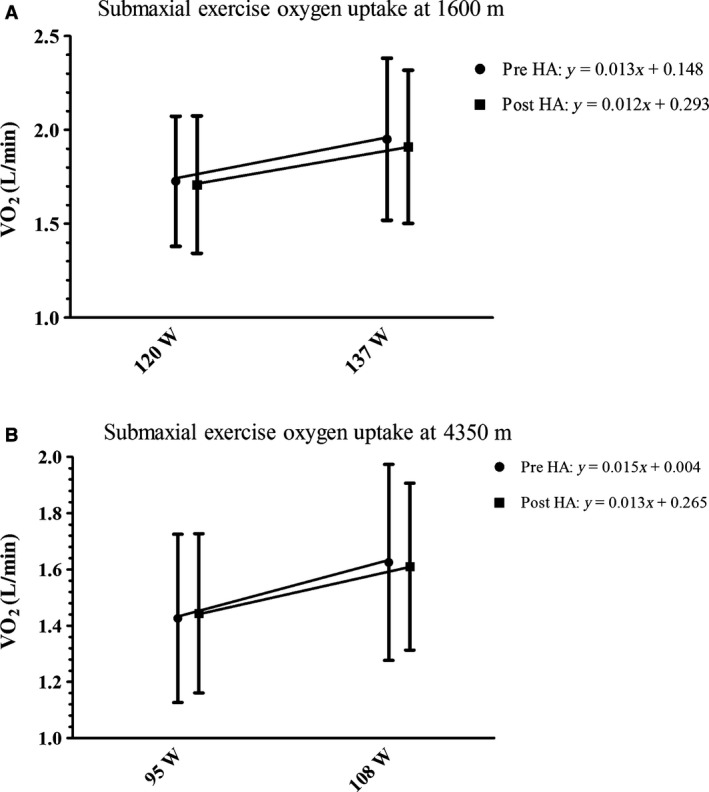
Illustrates submaximal exercise oxygen uptake at two different exercise intensities (indicative of exercise economy) and the mean slope and *y*‐intercept (A) at 120 ± 30 W and 137 ± 35 W at 1600 m; and (B) at 95 ± 23 W and 108 ± 26 W at 4350 m was not different from pre to postheat acclimation (HA).

Figure 4A and B shows the mean oxygen uptake (L/min) at low and moderate exercise intensities at 1600 m and 4350 m. At 1600 m, from pre‐to post‐HA, the mean oxygen uptake at 120 ± 30 W and 137 ± 35 W was not different (*P* > 0.05). The mean slope and mean *y*‐intercept determined from low‐to‐moderate exercise intensity were compared from pre‐to post‐HA (Fig. 4A). Neither the mean slope nor mean *y*‐intercept was different, indicating no change in exercise economy. At 4350 m, from pre‐to post‐HA, the mean oxygen uptake at 95 ± 23 W and 108 ± 26 W was not different (*P* > 0.05). When the mean slope and mean *y*‐intercept determined from low‐to‐moderate intensities were compared from pre‐to post‐HA, neither the mean slope nor mean *y*‐intercept was different, indicating no change in exercise economy (Fig. 4B). There was no difference in VCO_2_, VE, RER, HR, or RPE during submaximal exercise from pre‐ to post HA at 1600 or at 4350 m (*P* > 0.05).

## Discussion

We sought to evaluate whether heat stress can lower UCP3 expression and subsequently improve mitochondrial efficiency in murine myocytes. Further, in humans, we investigated if changes in cellular mitochondrial efficiency would result in improvement in exercise economy following a HA protocol and potentially be of benefit during exercise in a hypoxic environment. Our findings were: (i) in C2C12 myocytes, 24‐h of heat stress reduced UCP3 mRNA expression, thereby improving mitochondrial efficiency; and (ii) in humans, 10 days of HA induced characteristic changes in cardiovascular and thermoregulatory measures, however, submaximal exercise economy was not improved at 1600 m or 4350 m of altitude.

In this study, we demonstrated that heat stress reduced UCP3 mRNA expression and was accompanied by reduced mitochondrial uncoupling (that is, less energy “wasted” as thermogenesis during mitochondrial respiration). Furthermore, when cellular oxygen consumption was standardized to mitochondrial content, the oxygen consumption per mitochondrion was lower, indicating an improvement mitochondrial efficiency in the heat‐stressed cells. A reduction in mitochondrial respiration and UCP3 mRNA expression and endogenous proton leak helps to reduce heat production within the cell and can be thought of as a useful thermal adaptation in the face of thermal challenge. This adaptation may account for the reduction in basal metabolic rate with HA reported in some animal species (Cassuto 1968; McKechnie 2008).

We also found that heat stress increased gene expression of markers of mitochondrial biogenesis (PGC‐1*α*, NRF‐1 and TFAM) (Fig. 3A) which led to greater mitochondrial content (Fig. 2C) which has previously been reported by Liu and Brooks (2012). Liu and Brooks (2012) also observed heat‐stressed C2C12 myotubes had an increase in AMP‐activated protein kinase (AMPK), an enzyme that regulates PGC‐1*α*. Although AMPK was not measured in this study, it is likely that the increase in mitochondrial content and PGC‐1*α* in this study was due to greater AMPK from heat stress. The increase in PGC‐1*α* with simultaneous reduction in UCP3 may explain the increased mitochondrial content with concurrent decrease in peak mitochondrial respiration, as observed here.

While we observed an improvement in mitochondrial efficiency in our cellular experiments, these changes were not reflected as improved submaximal economy in heat‐acclimated humans. Following HA, while at 1600 m, submaximal exercise economy was not significantly different from pre‐HA steady‐state work at low or moderate exercise intensities. These responses were similar to those observed by Young et al. (1985) who reported a nonsignificant ~1% improvement in cycling economy at SL, following a 9 day HA protocol. In contrast, Sawka et al. (1983) reported significant improvements of ~6% and ~5% in treadmill walking economy following 6 and 10 days of HA, respectively. Shvartz et al. (1977) also reported that following 7 days of HA, exercise economy was significantly improved by ~13.5% and ~10.8% at low and moderate step exercise intensity, respectively. In this study, it is unclear why significant improvements in submaximal economy following HA were not observed. While the fitness level of our volunteers and our HA protocol was similar to that in the study by Sawka et al. (1983), we used a cycle ergometer, whereas volunteers from Sawka et al. (1983) walked on a treadmill. However, the different modes of exercise do not necessarily explain the difference in findings between our study and that of Sawka et al. (1983). In the study, by Shvartz et al. (1977) participants performed bench‐step exercise. Given the familiarization needed for repetitive bench‐stepping movement, the large improvements in economy observed by Shvartz et al. (1977) may be attributed to a training effect.

In this study, following HA, peak power output from the maximal exercise test increased 11 W, while VO_2peak_ stayed the same. Sawka et al. (1983) also observed an increase of 11 W (4%) in peak power output following HA. However, they also observed a 4% improvement in VO_2peak_, which indicates a potential training effect. It is unclear as to what contributed to the increase in peak power output in our study and whether this improvement was related to the HA protocol or other factors (improved lactate threshold, increase in fast‐twitch motor unit recruitment, etc.) but oxygen uptake and power output at VT and RCP remained unchanged from pre‐HA values. However, it should be noted that the increase in peak power output reported here was accompanied by a nonsignificant improvement of ~28 sec in 16‐km cycle time‐trial performance, previously reported (White et al. 2016), and therefore may not have “clinical” relevance.

In our cellular model, heat stress lowered UCP3 and improved mitochondrial efficiency, however, in the human model; following HA, exercise economy was not different. Previous animal (Gong et al. 2000; Vidal‐Puig et al. 2000) and human (Mogensen et al. 2006) studies without the use of heat stress as an intervention have investigated the role of UCP3 and mitochondrial efficiency, and whole body oxygen uptake. Gong et al. (2000) and Vidal‐Puig et al. (2000) reported UCP3 knockout mice had enhanced mitochondrial coupling and reduced mitochondrial respiration compared to wild‐type counterparts. However, both research groups did not detect lower whole body oxygen uptake in the UCP3 knockout mice in resting conditions. It was concluded that cellular changes as a result of UCP3 knockout does not affect whole body oxygen consumption (Gong et al. 2000; Vidal‐Puig et al. 2000), as observed in this study. In humans, Mogensen et al. (2006) reported that mitochondrial respiration and P/O ratio (ATP formation per oxygen used) from isolated mitochondria collected from muscle biopsies of trained individuals was not altered by lower expression of UCP3. Their results indicate that down‐regulation of UCP3 does not contribute to the improved submaximal economy. Therefore, even if HA reduced UCP3 in our volunteers, the findings from Mogensen et al. (2006) indicates UCP3 would not alter mitochondrial efficiency. Muscle samples collected from our volunteers would potentially clarify if heat‐induced reduction in UCP3 can enhance mitochondrial efficiency; we did not detect lower oxygen consumption at the systemic level, therefore, muscle biopsies may not be necessary.

We also did not see any changes in submaximal exercise economy at 4350 m (Fig. 4B). It was thought that the potential improvements in exercise economy resulting from HA would have benefit during exercise in a hypoxic environment, that is, cross‐tolerance. Gibson et al. (2015) used a 10 day controlled hyperthermic protocol and found that oxygen consumption at 40% and 65% VO_2peak_ was not lower after HA in normobaric hypoxia (FIO_2_: 12%, ~4400 m). Lee et al. (2016) also reported unaltered oxygen consumption at 50% VO_2peak_ after 10 days of HA in normobaric hypoxia (FIO_2_: 14%; ~3100 m). Our findings confirm those of Gibson et al. (2015) and Lee et al. (2016), despite the fact that our study was performed in hypobaric hypoxic conditions. As we did not observe improvements in submaximal economy at 1600 m, a lack of improved economy in hypobaric hypoxic conditions (4350 m) is not surprising.

This study is not without limitations. In the human experiment, individuals were intermittently heat stressed 2 h a day over a 10 day period, whereas cells were heat stressed continuously for 24 h. We also recognize that there may be different cellular responses between muscle cell‐lines and muscle samples collected directly from volunteers. However, the use of cell‐lines is common (Allen et al. 2005) when collecting tissue samples directly from participants is not feasible. It could also be said that residence at 1600 m (Albuquerque, NM) would result in acclimatization to this altitude. However, the P_a_O_2_ at 1600 m is ~95 Torr with a corresponding S_a_O_2_ of ~95%, thus we do not feel residence at this altitude confounded our results. There is always a potential for a training effect with repeated day of exercise training. However, trained individuals were recruited to reduce this likelihood. Furthermore, as VO_2peak_ remained unchanged from pre‐to post‐HA, thus we believe a training effect was unlikely.

In conclusion, this study demonstrates that heat stress induces cellular adaptations in myocytes leading to a reduction in UCP3 expression and improved mitochondrial efficiency. However, the improved cellular efficiency was not apparent at the systemic level, as submaximal exercise economy did not improve following HA at 1600 m or in conditions of hypobaric hypoxia (4350 m).

## Conflict of Interest

No potential conflicts of interest are disclosed.
